# Salivary Calculus and Its Concomitants

**Published:** 1880-01

**Authors:** George Watt


### THE DENTAL REGISTER.

VOL. XXXIV.] JANUARY, 1880. [No. 1.

### SALIVARY CALCULUS AND ITS CONCOMITANTS.

BY GEORGE WATT, M. D., D. D. S.

*Read before the Ohio State Dental Society.*

When, in the present state of civilization, the olfactory nerves are detailed as detectives, individuals are found bearing to society the relation of lively corpses, who walk erect, as it were, to save funeral expenses. Their accompanying odors suggest an escape from the charnal house, or the "stiff room," of the anatomist. And yet, these persons will smile in your face, their snaggle-toothed mouths suggesting a cage of every unclean and hateful bird, not excepting even the gormandizing buzzard. They will talk you deaf in hollow tones, which testify that "their throat is an open sepulcher." And the energy with which they transmit and maintain disease in every tissue of their own bodies, from cell to cerebrum, proves that "the poison of asps is under their lips."

But what is to be done about all this? And who shall do it? These victims are our brethren and sisters, our sons and daughters, our wives and ourselves. So prevalent, so common is this condition, that it has come to be regarded as an essential in civilization, and its victims go into society without a thought of their unpresentable state; or at most, they attempt to disguise their stench by something which smells still stronger.

The phenomena pertaining to these victims are oftener noticed by dentists than by physicians, perhaps, because the former, from the nature of their practice, are longer and closer in contact with their patients; and further, because one of the leading local manifestations of this condition is recognized as pertaining to dental, rather than to general surgery. I refer to the morbid state popularly known as scurvy of the gums, with deposit of salivary calculus, or tartar.

There is constant warfare between vital force and chemical force. The great trial, ever in progress in the Court of the Universe, may be entered on the docket, as Affinity versus Vitality. When vitality gives up the contest with reference to any individual life, we are familiar with the vengeful activity with which the plaintiff crushes and grinds his victim; and, fast or slow, as the case may be, like the mill of the gods, he "grinds exceedingly fine."

But, to drop metaphor, it is a mistake to suppose that chemical affinity triumphs over vital force only after the death of the individual. For it is well recognized by pathologists that vitality is often so much enfeebled that elements, and even compound radicals, are separated from tissues during the life of the patient, when, in obedience to their affinities, as modified by surrounding circumstances, the remaining elements form new compounds, which may be either healthful or hurtful to the patient. To mention many illustrations of this fact would prove too tedious for our present purpose; but take a familiar case, such as the part played by sulphur, both in health and disease, in the human constitution. A trace of it seems to be necessary to the formation of the protein compounds, such as albumen, fibrine, etc. While vital force is at par, all is peace, both with this element and the tissues it helps to form; but let vitality be depressed, till affinity is able to overcome its influence, oxygen being usually the first to make new inroads, the various elements seek new combinations, and one compound formed under such circumstances, is *sulphuretted hydrogen*—a poisonous acid, gaseous with ordinary pressure—which is capable of destroying the functions and even the life of the red corpuscles, by its sulphur taking their iron, thus rendering them unable to give force for organic life. When the sulphur thus takes iron, it lets go the hydrogen, which may combine with the phosphorus of the protein compounds, forming another poisonous, gaseous acid, as deleterious as the one just described; or the hydrogen, thus liberated, or that liberated in any way, may, and generally does, combine with nitrogen, forming ammonia—an alkali less poisonous, perhaps, than the acids above referred to, but still capable of great mischief, when thus disseminated in the blood. We thus see that hydrogen, in seeking new combinations, forms two acids and an alkali. Any one of these may be formed, or all may result simultaneously; and in such case, the well known mutual affinity of acids and alkalies is asserted, and combination takes place. These hydrogen compounds are all gaseous at ordinary temperatures, and each has a highly offensive odor. Of course, then, a mixture of all three can not fail to highly offend the olfactories; but when free, their odors, singly or together, is mild, compared with that resulting from their combinations. An intense stink results from the combination of either of these acids with ammonia.

I think every member of this society can recall cases in which the breath, the perspiration, or both have been loaded with one or more of these hydrogen compounds. This ought not to be. In perfect health it is probable that all the ammonia given off should escape by way of the kidneys. When given off by the respiratory organs, the patient is dying to the extent manifested by this putrefactive process. He may object to cremation and refuse to be buried; but he is dead, nevertheless, to the full extent of the stink.

When this condition has been reached, the constitution of the patient has lost much of its resisting power, and degeneration of tissue rapidly results. In the organs most liable to inflammatory action, this degeneration of tissue is likely to manifest itself earlier than in others; but degeneration, in one of its degrees, at least, is likely to be found throughout the entire system.

Several varieties of degeneration are recognized by pathologists; for instance, the fibrous, the granular, the fatty and the calcareous. In dental surgery, it is probable the granular is oftener noticed than the others, though I have seen all four well defined in the same mouth. It is not proposed to consider the nature and consequences of each or all of these varieties of degeneration; but the aim is to notice a single result and its concomitant circumstances, as they are brought to the notice of the dental surgeon, in the morbid condition in which there is degeneration of the gums and sockets, with deposit of salivary calculus, or tartar.

It is not necessary here and now to give an analysis of tartar. That it is composed of lime salts, with organic matter containing nitrogen, is enough to know for the present purpose.

It is well known to every member that carbonate of lime (chalk, marble-dust, etc.) is not soluble in water, and yet, in this vicinity, and in much of our country, this salt is abundantly dissolved in the water of our wells and springs. It is also well known that carbonic acid (a gas, ordinarily,) is soluble in water to a moderate extent. When thus dissolved, it is no longer gaseous, but has assumed the form of a liquid. The water of our wells and springs is nearly saturated with this gas, which may be removed from it in various ways. When the water is heated, the acid escapes as a gas; and when thus treated, several phenomena occur. One—important to be noticed here—will be better understood after stating that water, thus holding carbonic acid in solution, is able to dissolve carbonate of lime, as well as some other salts. When the acid is expelled by heat, the lime salts are precipitated, because the water, unaided, can not hold them in solution. This is constantly illustrated by the conduct and condition of the family tea-kettle. But, if instead of removing the carbonic acid by heat, we separate it by an alkaline base, the lime salt is precipitated all the same. A solution of soda or potash will give this result, as will also ammonia, whether gaseous or in solution. Any of you can demonstrate this by the most simple experiments.

Carbonic acid has constant access to the saliva, which dissolves it to the point of saturation. When thus charged, the saliva is able to hold the normal quantity of lime salts in solution; but if, by any process, the carbonic acid is removed, these salts are precipitated.

Now, take into consideration the condition of the putrefying patient above described, and you will find that ammonia is present in the buccul fluids. This, an alkali, takes the gas, an acid, thus forming carbonate of ammonia, leaving the fluids no longer able to hold the lime salts in solution, and they are precipitated in the form of tartar. As the salts are being precipitated, they entangle mucus (and perhaps other organic matter), and, as this putrefies, more ammonia is formed, giving thus a second source of mischief from this deleterious gas.

To break up the tendency to deposit tartar in any constitution, then, it is necessary to obviate the tendency to the formation or liberation of ammonia in the mouth. I say *formation*, or *liberation*, for it may be present in the mouth from either or both of these sources. It may be given off as a secretion, or rather as an excretion,from the salivary glands; and it may be,and indeed, always is, formed by the putrefaction of the organic matter contained in the salivary calculus. The porosity of tartar insures the absorption of any or all liquids placed in contact with it; and the condensation of gases, and, therefore, oxygen, is as well assured from the same cause. Thus condensed, the oxygen and nitrogenous matter are brought more thoroughly within the range of affinity, and, by taking one constituent to itself, the oxygen liberates the other elements, which, in seeking new associations, always give hydrogen in combination with nitrogen, which is well known as ammonia. And, as long as this is formed, so long will the lime salts be precipitated from the saliva, because the dissolving agent, carbonic acid, is taken by the ammonia, and the well known phenomenon, the deposit of tartar, will be recognized.

It follows, then, that even the most careful and thorough removal of the tartar is not all that is needed by the patient. True, this removal will do very much to prevent the presence of ammonia, but it can not do all. The sensible physician would not expect to cure typhoid fever merely by even the most careful removal of the patient's excrements from the sick-room ; but he certainly would not attempt the cure by allowing them to accumulate and remain therein from day to day.

As long as the fluids of the mouth are neutral in their reactions, there can be no deposit of tartar. But our profession, in recognizing dental caries as our opprobrium, and bearing constantly in mind that the immediate cause of caries (of each variety), is to be found among the acids, have, thoughtlessly, I fear, settled down to the opinion that, if a neutral state of the buccal fluids promises exemption, an alkaline condition gives still stronger promise of immunity. But it is doubtful if a greater mistake has ever been entertained by us.

In the local treatment for the condition described, it is necessary to carefully and thoroughly remove, not only the tartar, but also the remains of *debris* or the degenerated periosteum, as far into the sockets as this degeneration is complete; for its restoration to health after it has reached even granular degeneration, is not practicable in our present state of scientific attainments. And right here the profession is much indebted to Dr. Riggs for so earnestly inculcuting thorough local treatment, as well as for the introduction of improved instruments for the removal of this morbid and degenerated matter. It is unfortunate that so many of our brethren rely more on recipes than on basal principles; and it is much easier to remember that the tartar and other dead matter should be scraped away, than it is to make proper diagnosis, and to prescribe such therapeutic and hygienic treatment as will lift the constitution above the morbid state that cannot fail, if continued, to cause deposit of tartar, absorption of the gums and sockets, loosening and loss of the teeth, impairment of digestion, general failure of health, and shortening of life.

I feel confident that you will all agree that, after the period of youth has passed, a dozen teeth are lost by the morbid state under consideration for every one lost from other causes. This is the fact that makes the subject so ill important. And its great importance is the explanation of my conduct in devoting so many hours and days to its investigation, by both research and experiment. My persevering earnestness in persistently calling your attention to it, year by year, is also explained in the same way, as is the fact of my writing this while too ill to do so.

You all recognize and lament the condition I am describing. If, now, you recognize its immediate, as well as its predisposing causes, we are ready to suggest intelligent treatment. Short of such recognition, we grope in the dark, and our treatment is, at best, empirical. That there may be no tartar deposited, the saliva must remain saturated with carbonic acid. This cannot be when ammonia is formed, by putrefaction, within the mouth, nor when it is given off by the salivary glands. It follows, then, that the care of the general health is essential to the preservation of the teeth; and, when we are properly developed and cultivated, right here is where our profession is to be regarded as a branch of general medicine and surgery. How the knowledge necessary for the proper practice of the specialty is to be obtained, has nothing to do with this question, for when it has been reached, it forces recognition as certainly as does gravitation. Quacks and others, in both departments, may fall short of such attainments; and they may fail to be recognized; but they are not the profession. The general practitioner may point the finger of scorn at the ignorant dentist, who may be just what he calls him—a pretentious tooth-carpenter—and ask if we expected to be regarded as professional brothers, while such is a specimen of our craft. But we, in return, might inquire if they expect recognition from us while "the genuine Old Doctor Jacob Townshend," and "the aged minister whose sands of life are nearly run out," are general practitioners?

But to get rid of the deposit of tartar and the constitutional condition that gives rise to it:—

The system must be built up as thoroughly as possible, so that the vital force may "hold the fort" against the encroachments of affinity. And here, if not yourselves competent to prescribe, you will find it necessary to obtain the aid of the family physician; and if he and you are what you ought to be, you will be able to co-operate. And, as it is an alkali that takes the carbonic acid from the buccal fluids, and thereby causes the lime salts to be precipitated, we must try to get rid of this alkali—neutralize that which is already formed and present, as well as prevent the formation of more

After what has been already said, do you not all think of acids? and, if so, what acids? and how should they be used?

It is very common in this condition to prescribe the mineral acids, such as elixir vitriol, the dilute, sulphuric, nitric, or muriatic acids. At least, such course is very common with physicians, who seldom even take into consideration as an element or phenomenon of the disease, the deposit of tartar. As defective biliary secretion is usually present in this condition, these mineral acids often do good by stimulating the liver, as well as by neutralizing the alkaline condition of the secretions. There is but little choice among them —the elixir virtriol (aromatic sulphuric acid), having a slight advantage in taste, is often preferred. It may be given three timess day, in doses of ten to twenty drops, till the difficulty is removed.

But, in general, I prefer the vegetable acids, to be used as food or condiments. The citric acid (found in the lemon and elsewhere) is excellent, and at the same time so palatable, that the patient is all unconscious of the fact of taking medicine. And we all know that a stomach undisgusted will all the better perform its functions.

It is an axiom that "the best is the cheapest" but the cheapest is not always the lowest priced; fortunately, in the case before us, however, we find it so; for, to obviate this alkaline condition, acetic acid is the most difficult of all. Good vinegar is all that is needed to correct the morbid condition, so long as it is simple. If the patient's debility have continued till organic or other morbid changes have taken place, of course, these conditions must be met and treated according to their nature. But, by judicious use of vinegar (or pickles, if you please), the condition which deposits tartar need never be reached.

But now comes the question: "What did sin, this man or his parent, that he was born" to loose his teeth? In other words, we must never forget the force of heredity, in our consideration of this disease. If you know that parents have lost, or are loosing their teeth in this way, be doubly dilligent with the offspring. Point out the danger, instruct as to the preventive and remedical measures indicated, give assurance and confidence as to the certainty of relief, when the proper course is rigidly followed, and you will prove yourselves to be benefactors to your fellowmen; and when the great human race has been run, you shall be crowned as victors and the Judge will say, in presence of the assembled universe, "Ye did run well."

And now, brethren, this paper is not what I expected, far less what I hoped to give you. But till it was written, I felt that my professional work was unfinished. Now, I feel that it is done, but am sorry to be unable to add, well done. This has been prepared amid much weakness; but further postponement and abandonment had come to be synonymous terms; and so, here is the paper.

*Transactions, O. S. D. S.*

